# Progress in Assays of HMGB1 Levels in Human Plasma—The Potential Prognostic Value in COVID-19

**DOI:** 10.3390/biom12040544

**Published:** 2022-04-05

**Authors:** Michal Štros, Eva Volfová Polanská, Tereza Hlaváčová, Petr Skládal

**Affiliations:** 1 Institute of Biophysics of the Czech Academy of Sciences, 61200 Brno, Czech Republic; polanska@ibp.cz; 2 Department of Biochemistry, Faculty of Science, Masaryk University, 60177 Brno, Czech Republic; 451092@mail.muni.cz (T.H.); skladal@chemi.muni.cz (P.S.)

**Keywords:** HMGB1, plasma/serum, ELISA, EMSA, immunosensor, COVID-19

## Abstract

Extracellular HMGB1 protein is known to induce inflammatory responses leading to an inflammatory storm. The outbreak of the Severe Acute Respiratory Syndrome COVID-19 due to the SARS-CoV-2 virus has resulted in a huge health concern worldwide. Recent data revealed that plasma/serum HMGB1 levels of patients suffering from inflammation-mediated disorders—such as COVID-19, cancer, and autoimmune disorders—correlate positively with disease severity and vice versa. A late release of HMGB1 in sepsis suggests the existence of a wide therapeutic window for treating sepsis. Rapid and accurate methods for the detection of HMGB1 levels in plasma/serum are, therefore, of great importance for monitoring the occurrence, treatment success, and survival prediction of patients with inflammation-mediated diseases. In this review, we briefly explain the role of HMGB1 in the cell, and particularly the involvement of extracellular HMGB1 (released from the cells) in inflammation-mediated diseases, with an emphasis on COVID-19. The current assays to measure HMGB1 levels in human plasma—Western blotting, ELISA, EMSA, and a new approach based on electrochemical immunosensors, including some of our preliminary results—are presented and thoroughly discussed.

## 1. The Objectives of the Paper

The goal of the review paper is to outline and critically discuss current assays to measure HMGB1 levels in human plasma—Western blotting, ELISA and EMSA—with an emphasis on a new approach based on electrochemical immunosensors. To understand the rationale for the determination of HMGB1 levels in human plasma, we briefly summarize the role of HMGB1 protein in the cell and outside the cell (extracellular HMGB1), to support the idea of the functioning of the extracellular HMGB1 as a biomarker for survival and treatment success of patients with inflammation-mediated diseases, including COVID-19. This review paper also presents our results on the highly efficient pretreatment protocol for the removal of contaminants from human plasma prior to the HMGB1 assays, as well as preliminary data on the determination of HMGB1 protein by an electrochemical immunosensor.

## 2. HMGB1 Protein

**Figure 1:**
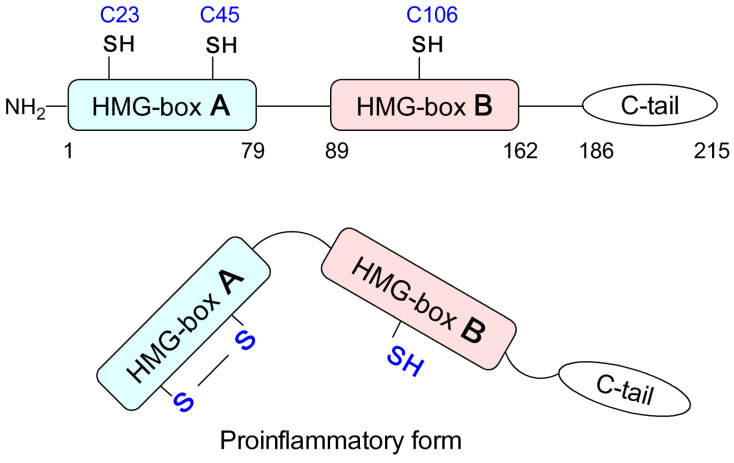
The structure and pro-inflammatory modification of HMGB1 protein. Mild oxidation of sulfhydryl groups of C23 and C45 within the HMG-box A results in a disulfide bond, converting the protein to a pro-inflammatory molecule. C, cysteine; C-tail, acidic C-terminus of HMGB1; SH, sulfhydryl group of cysteine.

The HMGB1 protein is a member of the highly conserved DNA-binding protein family, possessing a unique DNA-binding domain, the HMG-box (reviewed in [1]). There are four HMGB-type proteins in mammals (HMGB1-4). HMGB1 is a non-histone chromatin-associated protein with predominant nuclear localization, and it is ubiquitously and constitutively expressed in eukaryotic cells [2]. HMGB1 contains two HMG-boxes, A and B, arranged in tandem, and the highly acidic C-tail (Figure 1). HMG-boxes are not unique for the HMGBs proteins, and they can be found in a great number of biologically important proteins, including transcription and chromatin remodeling factors (reviewed in [1]). HMGB1 is indispensable for life, as revealed by the *Hmgb1-/-* knockout mice [3].

The DNA binding motif of HMGB1 is the HMG-box that binds the B-type DNA with no sequence-specificity and with a low affinity [1]. HMGB1 interacts with a great number of proteins, including transcription factors, such as p53/p73 and selective proteins of the Rel family ([1,2,4,5] and refs. therein). The above-mentioned binding abilities enable the HMGB1 protein to act inside the cell nucleus as a unique DNA chaperone, influencing multiple processes in chromatin, including transcription, replication, nucleosome sliding, V(D)J recombination, DNA repair, telomere homeostasis, and genomic stability (reviewed in [1,6]).

HMGB1 does not reside exclusively within the nucleus, but it can actively translocate to the cytoplasm through post-translational modifications, such as acetylation [7], ADP ribosylation, and redox state of cysteine 106 (reviewed in [1]). The protein can also be passively or actively released from cells [8,9]. The released (extracellular) HMGB1 has been extensively studied as a DAMP (Damage-Associated Molecular Pattern) protein alarmin, activating innate immunity via distinct receptors (reviewed in [10,11]). The redox states of three cysteine residues at positions 23, 45, and 106 are critical for the activity of HMGB1. While the all-thiol-HMGB1 can promote chemokine production and leukocyte recruitment, the disulfide-HMGB1 form can promote the release of pro-inflammatory cytokines. Fully oxidized HMGB1 is inactive [8]. Another post-translational modification of HMGB1 is acetylation, which is important for diminishing its association with chromatin and the subsequent release of HMGB1 to the extracellular fluid by cell stress or death ([7], reviewed in [1,12]).

Extracellular HMGB1, on its own or in complex with partner molecules (such as DNA, RNA, histones, nucleosomes, lipopolysaccharide (LPS), and interleukins IL-1α and IL-1β), exhibits pro-inflammatory, immuno-regulatory, and pro-tumor effects through autocrine and paracrine mechanisms (reviewed in [13]). HMGB1 is a late-stage inflammatory factor that is detectable after ~8 h upon stimulation of monocytes/macrophages by different exogenous factors, suggesting the existence of a wide therapeutic window for treating sepsis ([14] and refs. therein). 

## 3. HMGB1 Expression in Human Disease and Its Prognostic Value

The increased levels of HMGB1 in serum/plasma were observed in numerous inflammation-driven human diseases, such as autoimmune disorders (rheumatoid arthritis, systemic lupus erythematosus (SLE), and vessel vasculitis [12,15,16,17]) and cancer. It is of extreme interest to explore the prognostic and biomarker potential of HMGB1 in cancer since HMGB1 expression is frequently enhanced in advanced cancer stages and correlates with poorer prognosis in most human cancers ([18,19,20] and refs. therein). Recent data revealed that HMGB1 (also HMGB2 and HMGB3) seems to be a promising predictive biomarker for the prognosis, immunotherapeutic response, and immunotherapy target of multiple cancers [21].

## 4. HMGB1 and COVID-19

The outbreak of pneumonia COVID-19 by a novel, highly transmissible and pathogenic coronavirus SARS-CoV-2 at the end of 2019 has resulted in a huge health concern worldwide and has disrupted the health systems of the entire planet. The clinical course of COVID-19 varies substantially among patients. SARS-CoV-2 infection involves the following phases: (i) binding of the virus to cells and incubation, (ii) virus release and “cytokine storm”, and (iii) a massive lymphocyte infiltration associated with acute lung injury (reviewed in [22]). Binding of the spike protein of SARS-CoV-2 virus to the ACE2 (Angiotensin Converting Enzyme 2) protein on lung and intestinal cells leads to respiratory and homeostatic difficulties and brings about sepsis. Higher ACE2 expression, especially in lung epithelial cells, likely contributes to increased susceptibility and severity of SARS-CoV-2 infections [23]. SARS-CoV-2 or other virus-infected cells release endogenous DAMPs to alarm the environment about a loss of intracellular homeostatic balance [12,24]. One of the most extensively studied DAMPs is HMGB1 [25]. HMGB1 could play a key role in regulating the inflammatory response in COVID-19 by promoting SARS-COV-2 replication [24,26]. HMGB1 knockout protects cells from SARS-CoV-2-induced death, and the degree of protection was correlated to HMGB1 levels [27]. HMGB1 can also induce ACE2 mRNA expression in an AGER-dependent manner [28]. The genetic (using AGER siRNA) or pharmacological (such as glycyrrhizin, chloroquine, and hydroxychloroquine) inhibition of the HMGB1–AGER pathway can block ACE2 expression [28]. Interestingly, ACE2 overexpression has been reported to reduce HMGB1 [29], offering the hypothesis that a reduction in ACE2 induced by the virus would in turn increase HMGB1, thus contributing to the “cytokine storm” and the worst scenarios seen with COVID-19 infection [30]. Apart from HMGB1, S100A8/A9 (low molecular weight calcium binding proteins acting as alarmins) were also significantly enhanced, indicating that significant elevation of both S100A8/A9 and HMGB1 was associated with high mortality [26]. HMGB1 and interleukin-6 (IL-6) plasma levels at ICU admission were reported to be elevated compared with a healthy control, in both ICU non-survivors and ICU survivors [31]. Thus, in addition to HMGB1, other factors, such as different cytokines and alarmins, need to be observed in COVID-19 patients in order to develop effective tools for monitoring disease progression and outcome predictions.

In summary, the available data strongly indicate that progression from acute respiratory failure to sepsis in COVID-19 does correlate with the release of HMGB1 ([14,24,26,30] and refs. therein). HMGB1 may be a potential therapeutic target in severe cases of COVID-19 [30], and HMGB1 inhibitors can represent promising drug candidates for the treatment of patients suffering from the disease [28]. Thus, accurate measurements of HMGB1 in human plasma/sera may be a useful strategy to effectively tailor the disease [32]. It would be of interest to clarify whether (and if so, to what extent) different HMGB1 protein isoforms and/or *Hmgb1* gene polymorphisms (reviewed in [1]), could contribute to different progression and outcome in COVID-19 patients ([14,24,26,30] and refs. therein).

## 5. Methods for HMGB1 Determination

Monitoring of HMGB1 levels in plasma/serum may serve as a prognostic factor and biomarker for survival and treatment success of COVID-19 patients or other patients with inflammation-related diseases. Here, we would like to summarize the currently available methods (Western blotting, ELISA, and EMSA) for measuring HMGB1 levels in human plasma/serum, as well as briefly outline a principle of a new electrochemical assay for the determination of HMGB1 levels in human plasma that is currently under development in our lab.

## 6. Preparation of Plasma

HMGB1 levels in sera are significantly higher than in plasma due to HMGB1 release from cells when blood is allowed to clot [33]. Therefore, we recommend plasma, rather than serum, for reliable estimation of HMGB1 levels. For plasma preparation, blood is collected in EDTA or heparin solutions to prevent coagulation, followed by centrifugation of the samples (hemolytic samples are not suitable for ELISA). Plasma must be used for ELISA within 7 days when stored at 4 °C, or within 3 months when stored at −80 °C in small aliquots (liquid nitrogen is recommended for prolonged storage). Repeated freeze–thaw cycles should be avoided.

## 7. Factors in Serum/Plasma Affecting Accurate HMGB1 Determination

HMGB1 has a strong bipolar charge and is prone to interact with DNA, RNA, and numerous proteins, including IgG/IgM antibodies, lipids, and other factors in serum/plasma, effectively decreasing the measured HMGB1 levels [25]. HMGB1 levels detected by ELISA were significantly lower than those expected from immunoblotting results [34]. This was partially due to the binding of the IgG and IgM antibodies in sera/plasma in both healthy and ill individuals, which interferes with HMGB1 binding to anti-HMGB1 antibodies used in ELISA [34]. The interference was pronounced in diseases with a high prevalence of autoantibodies against HMGB1, such as in patients with autoimmune diseases ([35] and refs. therein). Other proteins (such as haptoglobin and cytokines) can also form complexes with HMGB1, making it inaccessible to the specific antibodies used for the detection of HMGB1 [36]. The incubation of recombinant HMGB1 with IL-1β, LPS, or specific antibodies can significantly reduce the amount of HMGB1 detected by ELISA, and treatment of the samples with perchloric acid (PCA) prior to ELISA can efficiently reverse this inhibition [36]. Pre-treatment of human plasma samples with PCA can efficiently and rapidly remove most of the contaminants (such as serum proteins, including IgG/IgM antibodies, lipids, and salts), masking the HMGB1 detection by a specific antibody and resulting in significantly higher amounts of HMGB1 in plasma samples from patients with septic shock compared to conventional ELISA [36]. The PCA pre-treatment can also overcome the problem of a relatively short half-life for HMGB1 isoforms (resulting from post-translation modifications, such as acetylation and oxidation) in patients if performed immediately after serum preparation. 

**Figure 2:**
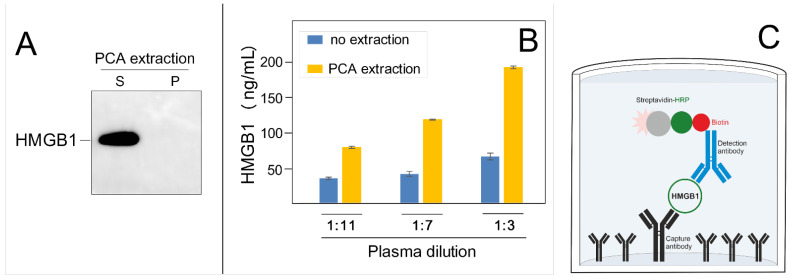
(**A**). Perchloric acid extraction of HMGB1 in human plasma. Left, PCA extraction of HMGB1 from human plasma (S, supernatant; P, pellet). Right, determination of HMGB1 by ELISA in plasma samples of patients before or after PCA extraction. A short protocol: one volume of human plasma was mixed in 1.5 mL Eppendorf tubes with 0.25 volume of 25% perchloric acid, vortexed, briefly centrifuged, and then neutralized with 0.5 volume of 1.5 M NaOH (relative to the volume of the plasma). Upon centrifugation, the supernatants (S) and pellets (P) were resolved on polyacrylamide gels, followed by transfer onto the PVDF membrane and detection of HMGB1 protein with specific antibody (W. blotting). (**B**). ELISA assay of HMGB1 levels in human plasma before and after PCA extraction. Human plasma, non-extracted, and PCA extracted, was diluted with PBS buffer as indicated, and the HMGB1 levels were then assayed by the sandwich ELISA. (**C**). Principle of the sandwich ELISA assay of HMGB1. A multi-well plate was coated with a polyclonal or monoclonal “capture” HMGB1 antibody. The plate was then used to immobilize HMGB1 from human plasma. The biotinylated “detection” HMGB1 antibody (a highly specific monoclonal antibody) was added to facilitate the subsequent detection using horseradish peroxidase (HRP)-conjugated streptavidin in the presence of a suitable substrate. Colorimetric detection is used to estimate HMGB1 levels when compared with the HMGB1-calibration curve.

Our recent experiments revealed that PCA-extraction of human plasma resulted in nearly 100% solubility of HMGB1 protein in the acid (Figure 2A), removing most of the contaminants (data not shown), demonstrating the feasibility of the PCA-extraction for the determination of HMGB1 protein in human plasma. The necessity to perform PCA-extraction before treatment of human plasma and before ELISA is directly supported by our preliminary ELISA experiments demonstrating that PCA-extracted human patient plasma contained 2–3 times more HMGB1 than the non-extracted human plasma (Figure 2B).

## 8. Western Blotting

Western blotting (W. blotting) enables the detection of HMGB1 immobilized on a nitrocellulose or a PVDF membrane. It can be also used to analyze different isoforms of HMGB1, as well as to quantify HMGB1 in serum/plasma samples. Typically, proteins from serum or plasma are first resolved by SDS-polyacrylamide gel electrophoresis, followed by electrophoretic transfer onto the membrane. The (albumin- or milk-blocked) membrane is then probed with a primary anti-HMGB1 antibody, washed, and incubated with a horseradish (HRP)-conjugated secondary antibody. The membrane is then washed and, in most protocols, incubated with an HRP substrate, providing a chemiluminescent product, resulting in a reasonably high signal [37].

Determination of HMGB1 in serum/plasma samples by W. blotting is laborious and only semi-quantitative. However, W. blotting can be used in combination with mass-spectrometry (MALDI-TOF) to analyze different isoforms of HMGB1. Although MALDI-TOF represents the most accurate method for basic research, it is expensive, time-consuming, and not available at the typical point of care. Currently, most HMGB1 measurements in human serum/plasma are performed by ELISA. Modification of ELISA using antibodies specific to distinct HMGB1 isoforms can provide a tool for the rapid diagnosis of different types of diseases.

## 9. ELISA

ELISA (Enzyme-Linked Immuno-Sorbent Assay) is a technique to used detect the presence of antigens in biological samples. ELISA, similarly to other types of immunoassays, relies on antibodies to detect a target antigen using highly specific antibody–antigen interactions. In an ELISA assay for HMGB1, the protein (antigen) is bound to a solid surface. This is usually performed via the use of a capture antibody immobilized on the surface. HMGB1 is then complexed into a detection antibody conjugated with a molecule amenable for detection, such as an enzyme or a fluorophore.

Sandwich ELISA is the preferably used format for HMGB1 detection in human plasma/serum (Figure 2C). This protocol requires two antibodies (referred to as matched antibody pairs) specific for different epitopes of the HMGB1 protein. The HMGB1 antibody (either monoclonal or polyclonal) is coated on the surface of the multi-well plate and used as a “capture” antibody for HMGB1. The biotinylated “detection” HMGB1 antibody (a highly specific monoclonal antibody) is added to facilitate subsequent detection using horseradish peroxidase (HRP)-conjugated streptavidin. The most used substrate for ELISA is 3,3′,5,5′-Tetramethylbenzidine (TMB). TMB produces a deep blue color during the enzymatic reaction; upon the addition of sulfuric acid, an intensive yellow color is generated and measured at 450 nm. The absorbance at 450 nm is proportional to the concentration of HMGB1. The concentration of HMGB1 in serum/plasma can be calculated by comparing the absorbances of the samples with the standard HMGB1 curve. Currently, modified versions of TMB substrate with higher sensitivity, stability, and lower background are available on the market. Several ELISA kits are currently available using TMB and very good quality of matched HMGB1 antibody pairs (the HMGB1 epitopes of the antibodies in the commercial kits are proprietary) to determine HMGB1 concentration in plasma/serum. The sensitivity of HMGB1 determination in ELISA assay can be significantly increased when luminol is used as a substrate for HRP, and the resulting light emission is read by a luminometer, where the intensity of the emitted light is proportional to the amount of HMGB1 in the well. Any decrease in the time procedure of the HMGB1 ELISA assay would be advantageous, since it currently relies on 60 min to overnight incubation with serum/plasma, and another ~3 h are necessary to perform the assay. ELISA kits are commonly available in a 96-well plate format.

## 10. EMSA

In addition to W. blotting and ELISA, Gaillard and co-workers developed an EMSA (Electrophoretic Mobility Shift Assay) protocol based on the high affinity of HMGB1 for hemicatenated DNA (hcDNA) [38,39]. The complexes formed between HMGB1 and ^32^P-labeled hcDNA were analyzed by polyacrylamide gel electrophoresis, followed by autoradiography [39]. The EMSA assay required the preparation of ^32^P-labeled hemicatenanes, PCA-extracted plasma/serum, and resolution of the HMGB1–hcDNA complexes by polyacrylamide electrophoresis. Although this method is very sensitive, it is laborious and time-consuming, and the hemicatenated DNA loops can interact not only with HMGB1 but also with HMGB2 and other DNA-binding proteins ([40,41,42,43]), making the method non-applicable for routine use. Cross-reactivity of this assay with HMGB2, and possibly other proteins, can also partially explain the detection of significantly higher amounts of HMGB1 in serum/plasma by EMSA as compared to the conventional PCA-ELISA [36,39].

## 11. Electrochemical Immunosensor

ELISA is currently the only method routinely used in clinics for the determination of HMGB1 levels in plasma/serum. Although ELISA can detect as little as 0.2–2 ng/mL of HMGB1 (depending on the nature of the ELISA substrate and the detection method—chemiluminescence vs. absorbance), a prolonged time is needed for the analysis (typically 3–4 h to overnight, depending on the ELISA kit). Furthermore, ELISA assays are relatively expensive, and accurate determination of HMGB1 levels is not reliable due to interference with plasma/serum contaminants. Therefore, other assays for rapid, accurate, and cost-effective determination of HMGB1 levels in plasma/serum could be of interest for clinical practice.

**Figure 3:**
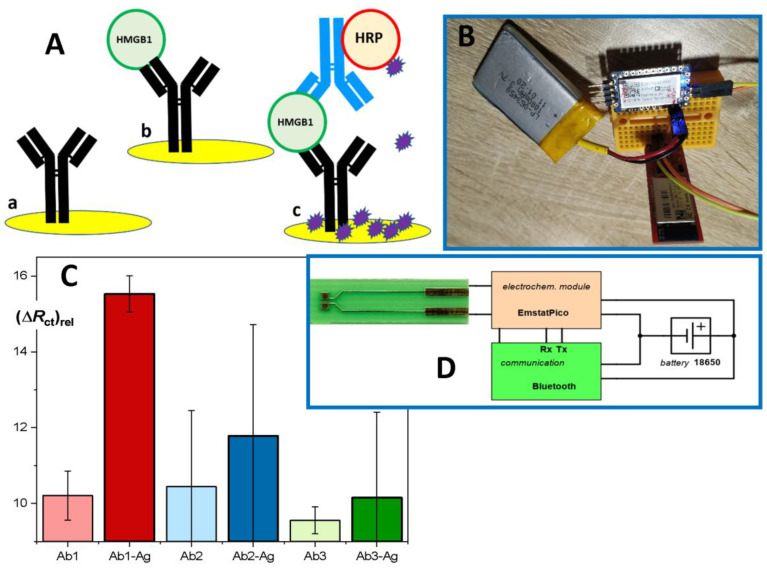
Concept of the HMGB1 immunosensor. (**A**) A gold electrode is modified with a capture antibody (a), and after incubation with the sample, the formed immunocomplex can be detected, not as a color change (ELISA) but as a change in impedance (b). An amplified response can be obtained using a sandwich format with secondary Ab-HRP conjugate, where the product of the enzyme label reaction precipitates on the electrode surface. (**B**,**D**) The electrochemical module EmstatPico (PalmSens) was linked to a Bluetooth module RN-42 (Sparkfun, or other similar) and communicated with a control smartphone (tablet or PC). The disposable sensor was based on printed circuit board technology (**D**, left). (**C**) Impedance changes depending on the choice of the capture antibody: light color—blank; dark color—positive samples of recombinant HMGB1 (Ab-Ag).

Here, we outline a new electrochemical immunosensor based on disposable and exchangeable sensing elements. The capture antibody is immobilized on a gold electrode (Figure 3A, (a)), and the interaction with HMGB1 (Figure 3A, (b)) can be directly followed as a change of impedance resulting from the blocking of the electrode surface after the formation of an immunocomplex. In this way, a rapid measurement consists of 2 min measurement of an initial impedance scan, 15 min incubation with the sample, and 2 min of repeated scanning of the impedance. The measuring intervals can be shortened if a single impedance value is obtained at one chosen frequency value. Using the response at 500 Hz as the evaluated signal, the limit of detection (based on signal/noise ratio 3:1) was near 2 ng/mL. This satisfies the clinical requirements. Substantially higher sensitivity is obtained using an additional step, i.e., secondary antibody conjugated to HRP (Figure 3A, (c)) resulting in an immuno-sandwich complex. Finally, colorimetric measurements typical for ELISA are replaced by amperometric or voltametric detection of oxidation of a substrate by HRP/H_2_O_2_; the well-known TMB can be used [44], though 4-chloro-1-naphthol (4-CN) provides an advantage of precipitating the product of the enzyme reaction (Figure 3A). 

The concept of the electrochemical immunosensor is based on convenient disposable electrodes made from the printed circuit board (PCB) material with gold-based sensing spots; this was already demonstrated as suitable for various electrochemical measuring techniques [45]. The adopted PCB technology allowed us to obtain large series of sensors (Figure 3D, photo at left) at very competitive costs [46].

Furthermore, after initial experiments with the design of a miniaturized potentiostat, we have adopted the highly integrated module Emstat Pico, which allows us to utilize several electrochemical techniques and can be easily connected through serial communication with USB-serial adapters, Bluetooth modules, and Arduino-like and Wi-Fi boards, such as ESP32. The testing set-up placed on a prototyping board was successfully utilized (Figure 3B), and the Li-polymer rechargeable battery allowed for up to 7 h of operation. The user is provided with MethodSCRIPT procedures for repeated use (https://www.palmsens.com (accessed on 1 April 2022)).

The performance of the immunosensor can be further improved using nanomaterials, such as colloid Au nanoparticles (NP) because of their large surface area, good biocompatibility, and suitability for many immobilization protocols ([47] and refs. therein). An alternative approach with a modifying layer of nanoparticles will be also tested, and higher sensitivity can be gained when immuno-complex formed within pores among neighboring NPs block the transport of a redox probe [48]. The success of the new method based on the electrochemical immunosensor heavily depends on the specificity of the monoclonal HMGB1 antibodies with high-affinity equilibrium constants; this is evident in Figure 3C, where Ab1 (HMGB1 N-terminal-specific) performed as expected, providing a high difference between the positive sample and blank. On the other hand, the alternative antibodies Ab2 and Ab3 failed and resulted in highly fluctuating and non-specific responses due to the low stability of the formed immunocomplexes and weaker binding of the secondary labeled antibody. The signal amplification currently relays on native horseradish peroxidase; its Prussian blue nanocrystal mimics can increase the robustness and stability of the label [49]. In addition, an advanced version of the immunosensor will be constructed as an eight-channel sensing strip suitable for simultaneous analysis of a few standards and samples; a simple analog switch and appropriate extended sensor design are under development. Finally, we have summarized the merits and demerits of all the available assays of HMGB1 levels in human plasma in Table 1.

## 12. Conclusions

HMGB1 in plasma/serum is a useful biomarker of the occurrence of inflammation-related diseases, including cancer and COVID-19. While the total HMGB1 protein in plasma/serum is indicative of both cell death and immune cell activation, specific isoforms of HMGB1, such as hyper-acetylated and/or disulfide (mildly oxidized form), appear to be more informative biomarkers for multiple disorders, in combination with other biomarkers ([50] and refs. therein). Currently, the only technique available for characterizing HMGB1 post-translational modifications is mass spectrometry (MALDI-TOF). Although MALDI-TOF represents the most accurate method for basic research and can be used for the identification of specific HMGB1 isoforms, it is time-consuming, expensive, and not available at the typical point of care. Thus, the immunosensor technique, taking advantage of monoclonal antibodies against specific HMGB1 isoforms to detect HMGB1 in plasma/serum could be a prospective routine method (in combination with other methods and biomarkers) for monitoring the occurrence, treatment success, and survival prediction of patients with inflammation-mediated diseases, including COVID-19.

## Figures and Tables

**Table 1:** Merits and demerits of HMGB1 assays.

Methods	Sensitivity	Specificity	Time	Laboriousness	Cost
Western blotting	++	++++	+++	+++	++
ELISA	+++ *	++++	++	++	++
EMSA	++++	+	+++	++++	+++
Immunosensor	+++	++++	+	+	+

Description: + low, ++ medium, +++ high, ++++ very high. * The sensitivity can be significantly enhanced upon chemiluminescent detection.
